# Genetic and Phenotypic Virulence Potential of Non-O1/Non-O139 *Vibrio cholerae* Isolated from German Retail Seafood

**DOI:** 10.3390/microorganisms11112751

**Published:** 2023-11-11

**Authors:** Quantao Zhang, Thomas Alter, Eckhard Strauch, Jens Andre Hammerl, Keike Schwartz, Maria Borowiak, Carlus Deneke, Susanne Fleischmann

**Affiliations:** 1 Institute of Food Safety and Food Hygiene, School of Veterinary Medicine, Freie Universität Berlin, Königsweg 69, 14163 Berlin, Germany; 2 Department Biological Safety, German Federal Institute for Risk Assessment, Diedersdorfer Weg 1, 12277 Berlin, Germany; eckhard.strauch@bfr.bund.de (E.S.); jens-andre.hammerl@bfr.bund.de (J.A.H.);

**Keywords:** non-O1/non-O139 *Vibrio cholerae*, seafood, genetic and phenotypic characterization, virulence potential, antimicrobial resistance

## Abstract

Non-O1 and non-O139 *Vibrio cholerae* (NOVC) can cause gastrointestinal infections in humans. Contaminated food, especially seafood, is an important source of human infections. In this study, the virulence potential of 63 NOVC strains isolated from retail seafood were characterized at the genotypic and phenotypic levels. Although no strain encoded the cholera toxin (CTX) and the toxin-coregulated pilus (TCP), several virulence factors, including the HlyA hemolysin, the cholix toxin ChxA, the heat-stable enterotoxin Stn, and genes coding for the type 3 and type 6 secretion systems, were detected. All strains showed hemolytic activity against human and sheep erythrocytes: 90% (*n* = 57) formed a strong biofilm, 52% (*n* = 33) were highly motile at 37 °C, and only 8% (*n* = 5) and 14% (*n* = 9) could resist ≥60% and ≥40% human serum, respectively. Biofilm formation and toxin regulation genes were also detected. cgMLST analysis demonstrated that NOVC strains from seafood cluster with clinical NOVC strains. Antimicrobial susceptibility testing (AST) results in the identification of five strains that developed non-wildtype phenotypes (medium and resistant) against the substances of the classes of beta-lactams (including penicillin, carbapenem, and cephalosporin), polymyxins, and sulphonamides. The phenotypic resistance pattern could be partially attributed to the acquired resistance determinants identified via in silico analysis. Our results showed differences in the virulence potential of the analyzed NOVC isolated from retail seafood products, which may be considered for further pathogenicity evaluation and the risk assessment of NOVC isolates in future seafood monitoring.

## 1. Introduction

*Vibrio cholerae* is a water-borne, Gram-negative bacterium found in aquatic ecosystems worldwide [1]. The *V. cholerae* serogroups O1 and O139 are considered to be human pathogens that lead to epidemic and pandemic cholera outbreaks. Those two serogroups act as the causative infectious agents of the intestinal disease cholera by producing cholera toxin (CTX) and toxin-coregulated pilus (TCP) as their main virulence factors [2,3].

However, the species *V. cholerae* is divided into more than 200 serogroups. Serogroups other than O1 and O139 are collectively referred to as non-O1/non-O139 *V. cholerae* (NOVC). In contrast to O1 and O139 *V. cholerae*, whose virulence mechanisms and metabolic pathways have largely been clarified [4], only limited knowledge is available regarding the virulence factors implicated in the pathogenicity of this large NOVC cluster. Therefore, in recent years, scholarly attention has been directed to further exploration of the pathogenic ability of NOVC.

NOVC infection history can be traced back to the 1990s, with several cases reported in South America and Southeast Asia [5,6]. Since then, virulence-associated factors have been identified in NOVC, including hemolysin HlyA, hemagglutinin protease HapA, repeats-in-toxin (RTX) toxins, sialidase Nan, heat-stable enterotoxin Stn, and type 3 (T3SS) and type 6 (T6SS) secretion systems [7]. Currently, there are several reports of NOVC infections, such as case reports and literature reviews [8]. The U.S. Centers for Disease Control and Prevention (CDC) reported 52 NOVC infection cases in the United States from 1998 to 2014. Seafood consumption was attributed to 89% of these cases. In all cases, symptoms resulted in diarrhea with abdominal cramps and nausea. Only 9% of patients had bloody stools. Overall, 38% of affected people were hospitalized, and none of them died. Due to the self-limiting disease process, it is currently assumed that there are around 100 cases per year, with further unreported and undiagnosed cases likely occurring [9]. In Europe, NOVC-associated cases were also reported [10]. In Germany, 30 NOVC cases were registered between 2003 and 2020, of which 17% caused gastroenteritis after oral ingestion [11].

Seafood consumption is an important source of NOVC infections. In Germany, NOVC are detected in approximately 6% of seafood products offered in retail markets [12]. However, studies focusing on NOVC strains isolated from seafood have previously been rather generally compared to isolates from patients and the environment. Ottaviani et al., 2009, analyzed 17 NOVC strains from different seafood sources. They found that all strains were positive for *hlyA* (Hemolysin encoding gene, El Tor biotype variant) [7], while the CTX phage (the cholera toxin encoding island, which contains *ctxA*, *ace*, *zot*, *rstA*, and *rstB*) was absent. Their virulence was further confirmed using Vero cells and an-mouse model [13]. The virulence potential of NOVC isolated from many kinds of seafood was also reported in other studies [14,15]. However, those studies paid much attention to the characteristics of pandemic strains such as CTX phage presence and hemolytic activity, while other genes related to virulence, regulation, and colonization were not addressed. Nevertheless, those genes might also contribute to the virulence performance of NOVC [7].

In addition to the presence of virulence factors, antimicrobial resistance (AMR) is an important trait [16]. Three mechanisms were identified as enabling *V. cholerae* to escape antibiotic treatment: point mutations of chromosomal genes affecting resistance development, efflux systems, and drug degradation enzymes. Efflux systems and drug degradation enzymes are physically linked to mobile genetic elements (MGEs), such as integrating conjugative elements (ICEs) and plasmids [17]. There are several commonly reviewed AMR related genes in NOVC, including *sul1/2* (sulfonamides resistance) and *strA/B* (streptomycin resistance), *int* (integrons of MGE), *setR*, *dfr* (dihydrofolate reductase), and *qnrVC* (quinolone resistance) [18,19,20]. Due to the mobile and interchangeable nature of MGEs, aquatic environments might become reservoirs for AMR [21].

Due to the limited number of cases, self-limiting disease, and unclear pathogenic properties, NOVC strains have not been included in current food safety standards by the U.S. Food and Drug Administration (FDA) or the European Food Safety Authority (EFSA) [22,23]. Therefore, the aim of this study is to characterize the virulence and antibiotic resistance potential of seafood-associated NOVC from a German market by analyzing different phenotypic and genotypic traits.

## 2. Materials and Methods

### 2.1. Strain Collection

In this study, a collection of 63 NOVC strains from German retail seafood was provided by the “Consultant Laboratory for *Vibrio* spp. in Food” hosted by the German Federal Institute for Risk Assessment (BfR). The strains originated from food matrices collected and investigated between 2014 and 2019, in accordance with two International Organization for Standardization methods, namely ISO/TS 21872-1/2007 and 21872-1/2017. In addition, two *V. cholerae* O1 El Tor strains, namely serotype Ogawa (DSMZ 100200) and serotype Inaba (DSMZ 106276), were included as positive controls. Further information on bacterial strains is given in Table S1. Prior to analysis, isolates were cultivated on lysogeny broth (LB) agar (Millipore, Merck, Darmstatd, Germany) for 18–24 h at 37 °C. Subsequently, a single colony was selected and cultivated overnight in LB medium (Millipore, Merck, Darmstatd, Germany) for 18 h at 37 °C. Strains were stored, based on the manufacturer’s instructions, at −80 °C in CRY80-tubes using 1 mL of the overnight culture (Mast Diagnostics, Reinfeld, Germany).

### 2.2. Whole-Genome Sequencing and Bioinformatics Analysis

All 63 NOVC strains were subjected to whole-genome sequencing (WGS). DNA was extracted from liquid overnight LB culture using the MasterPure™ DNA Purification Kit (Lucigen, Biozym Scientific, Oldendorf, Germany). DNA was quantified using the Qubit 2.0 fluorometer (Life Technologies, Darmstadt, Germany). Library preparation from genomic DNA was performed using the Nextera DNA Flex library preparation kit (Illumina, Inc., San Diego, CA, USA). Paired-end sequencing was performed via a MiSeq benchtop sequencer (Illumina, Inc., San Diego, CA, USA) in 2 × 300 cycles using the MiSeq Reagent Kit v3 (Illumina, Inc., San Diego, CA, USA). The AQUAMIS Pipeline (version 1.3.7) [24] was used for assembly and quality control. Raw reads were trimmed using fastp (version 0.20.1) [25] and assembled using shovill (Seemann 2020, version 1.1.0, https://github.com/tseemann/shovill (accessed on 12 October 2022)). All samples passed the quality criteria, as implemented in AQUAMIS; therefore, the samples showed sufficient base quality and coverage depth. The assemblies’ genome length (3,907,054 to 4,290,325 bp) and GC content (47.1% to 47.7%) were in their expected ranges, and there were no signs of contamination (https://bfr_bioinformatics.gitlab.io/AQUAMIS/report_test_data/assembly_report.html#thresholdt (accessed on 12 October 2022)).

In order to identify the phylogenetic relationships between the strains, core genome multilocus sequence typing (cgMLST) was employed using the chewieSnake pipeline (version 3.1.1) [26] and by applying the PubMLST cgMLST scheme for *V. cholerae* [27]. In total, the cgMLST schema includes 2443 loci. In all strains, 97–99% of the cgMLST loci could be identified. Four clinical NOVC sequences (AM-19226, MZO-2, MZO-3, and VN-300) and six O1/O139 pandemic strains (2010EL-1786, 2011EL-1271, 2012EL-2176, FJ147, Inaba G4222, and MO10) were included to draw conclusions about possible relatedness. A minimum spanning tree was inferred using grapetree [28] and visualized using Geneious software (v2022.1.1).

The presence or absence of virulence-associated genes for all strains was confirmed via Geneious software (v2022.1.1) using the nucleotide BLAST algorithm (medium sensitivity, default setting parameters) based on a match to the reference sequence with an identity of between 80 and 100%. The *V. cholerae* O1 serovar El Tor FJ147 (NZ_CP009041, NZ_CP009042), the *V. cholerae* O139 serovar MO10 (NZ_CP060094, NZ_CP060095), the environmental strain O1 Env390 (NZ_CP013013, NZ_CP013014), and the WGS of the available clinical T3SS positive NOVC strains AM-19226, MZO-2, MZO-3 [29], and VN-300 [7] were included as positive controls for virulence marker determination.

### 2.3. Hemolytic Test

For hemolytic tests, blood agar based on Mueller–Hinton agar (Oxoid GmbH, Wesel, Germany) supplemented with 5% human or sheep erythrocyte were prepared. Human blood was taken from healthy volunteers via venipuncture. The blood was collected using blood collection tubes containing dipotassium salts of ethylene diamine tetra acetic acid (K2EDTA) for use as an anticoagulant (DB Medical, Eysins, Switzerland). Defibrinated sheep blood was ordered from Thermo Scientific Oxoid, Landsmeer, Netherlands. Erythrocytes were washed three times using 0.01 M phosphate-buffered saline (PBS) buffer (Medicago, Uppsala, Sweden). PBS was removed as a supernatant after centrifugation (1000× *g* at 4 °C for 5 min). After washing, the isolated erythrocytes were compacted via a final centrifugation step at 1000× *g* for 10 min at 4 °C before being added to the agar.

Hemolytic tests were performed by inoculating 5 μL of LB overnight culture of *V. cholerae* strains in triplicate on the prepared blood agar plates. After 24 h of incubation at 37 °C, hemolytic activity was visually assessed, and the diameter of the complete hemolytic zone (beta hemolysis) was determined.

### 2.4. Biofilm Formation

The biofilm formation assay was performed using the method described by Mahoney et al., 2010 [30], albeit with minor modifications. In total, 3 mL of each of the LB overnight culture of *V. cholerae* strains was centrifuged at 5000× *g* for 3 min, and the pellet was resuspended in 500 µL PBS. The bacterial cell density was employed to the optical density (OD) of 0.7 at 588 nm. *V. cholerae* cells (125 µL) were then inoculated in 125 μL of LB broth in a 96-well clear polystyrene, non-treated, and flat-bottom microplate (Corning, New York, NY, USA). After 48 h of incubation at 25 °C, LB media was carefully removed via pipetting, and the plates were washed twice, using 300 μL of PBS buffer per well. The remaining biofilm was dried overnight at room temperature. For biofilm staining, 275 μL of 0.1% crystal violet solution (*w*/*v* in distilled water) was used. After 1 h of incubation, crystal violet was carefully removed via pipetting, and the stained complex was washed three times using sterile water and dried at room temperature for 1 h. The stained biofilm was dissolved in 300 μL of 33% acetic acid (*w*/*v* in distilled water) for 30 min. The OD of the staining solution was measured at 595 nm and normalized to the absorbance of LB as a negative control. According to OD595 absorbance, *V. cholerae* strains were assigned to four groups due to their biofilm formation ability: none (OD < 0.5), weak (OD 0.5–1.0), medium (OD 1.0–3.0), and strong biofilm formation (OD > 3.0). For all the strains tested, three biological replicates were analyzed, with each having ten technical replicates.

### 2.5. Serum Resistance

Serum resistance tests were performed according to a method previously described by Bier et al., 2013 [31]. In total, 12 µL of LB overnight culture was transferred in 600 µL of LB broth and incubated at 37 °C for 5–6 h, and 2 µL per well was transferred to 96-well microplates containing gradient mixtures consisting of 100 µL of human serum (pooled from healthy volunteers) and peptone–glucose broth (1% glucose, 0.0075% bromothymol blue, 1% peptone, 0.5% NaCl, pH 7.4). The blood serum concentrations were 0%, 10%, 20%, 40%, and 60%, respectively. Plates were incubated at 37 °C for 24 h, and the serum resistance was examined based on a color shift from blue to yellow, indicating metabolic activity via glucose fermentation. In each 96-well plate, a positive control of *Escherichia* (*E*.) *coli* K-12 + pKT107 (RS228) carrying the serum resistance plasmid and a negative control *E. coli* K-12 − pKT107 (DSMZ423) was used. Bacterial serum resistance was classified as none (bacterial growth was below 20% serum), weak (bacterial growth in up to 20% serum), medium (bacterial growth in up to 40% serum), or strong (bacterial growth in 60% serum). Three biological replicates were performed for all the tested strains.

### 2.6. Motility Determination

To determine the motility of *V. cholerae* isolates, a soft agar plate assay was performed based on the method of Li et al., 2022 [32], albeit with modifications. In brief, 1 µL of overnight LB broth cultured *V. cholerae* strains was injected directly into fresh 0.3% LB agar plates. The diameters of the motility zones were measured after 24 and 48 h of incubation at both 25 °C and 37 °C. For each strain, 6 to 9 replicates were performed. Motility was classified based on the diameter of the motility zone on the plate: none (0 cm), weak (0–4 cm), medium (4–8 cm), or strong (≥8 cm).

### 2.7. Antimicrobial Resistance Evaluation

Both in silico and in vitro approaches were employed to verify the antimicrobial resistance (AMR) pattern of all tested strains. Two online search engines, namely ResFinder (v4.1) [33] and Resistance Gene Identifier (v5.2.1) from the Comprehensive Antibiotic Resistance Database [34], were used for the screening of AMR-related genes with default settings. Moreover, a collection of recently identified AMR genes in NOVC was examined via Geneious software: the multidrug and toxic compound extrusion (MATE) efflux system (*vcmA*, *vcmB*, *vcmD*, *vcmH*, *vcrM*, and *vcmN*), the ATP binding transporter (vcaM), and the conserved region of SXT/R391 ICE (SXT/R391) [18,19,20].

As for the in vitro tests, antimicrobial susceptibility testing (AST) of *V. cholerae* was performed via broth microdilution based on the guidelines of the Clinical and Laboratory Standards Institute (CLSI) M07, 11th ed. M07-A10 [35,36]. We used a harmonized European panel of antimicrobials combined in a commercial plate format (EUVSEC, ThermoScientific, Meerbusch, Germany) containing 15 antimicrobial substances of 12 antimicrobial classes: ampicillin (AMP), azithromycin (AZI), cefepime (CEFEPI), cefotaxime (FOT), ceftazidime (TAZ), and clavulanic acid (CLA), as well as in combination with FOT (TAXCLA) or TAZ (TAZCLA), cefoxitin (FOX), chloramphenicol (CHL), ciprofloxacin (CIP), colistin (COL), ertapenem (ERTAPE), gentamicin (GEN), imipenem (IMIPEN), meropenem (MERO), nalidixic acid (NAL), sulphamethoxazole (SMX), temocillin (TEMOCI), tetracycline (TET), tigecycline (TGC), and trimethoprim (TMP). Antimicrobial substances were used in concentration ranges described in the European Commission implementing decision 2020/1729/EU [37]. The *E. coli* isolate ATCC 25922 was used as a quality control and reference strain during AST. The minimal inhibitory concentration (MIC) values of all strains are listed in Table S2. Multidrug resistance was defined based on combined resistance to ampicillin (AMP), cefotaxime (FOT) and/or ceftazidime (TAZ), and ciprofloxacin (CIP) (der KRINKO, 2012) [38].

## 3. Results

### 3.1. Genetic Characterization

**Figure 1:**
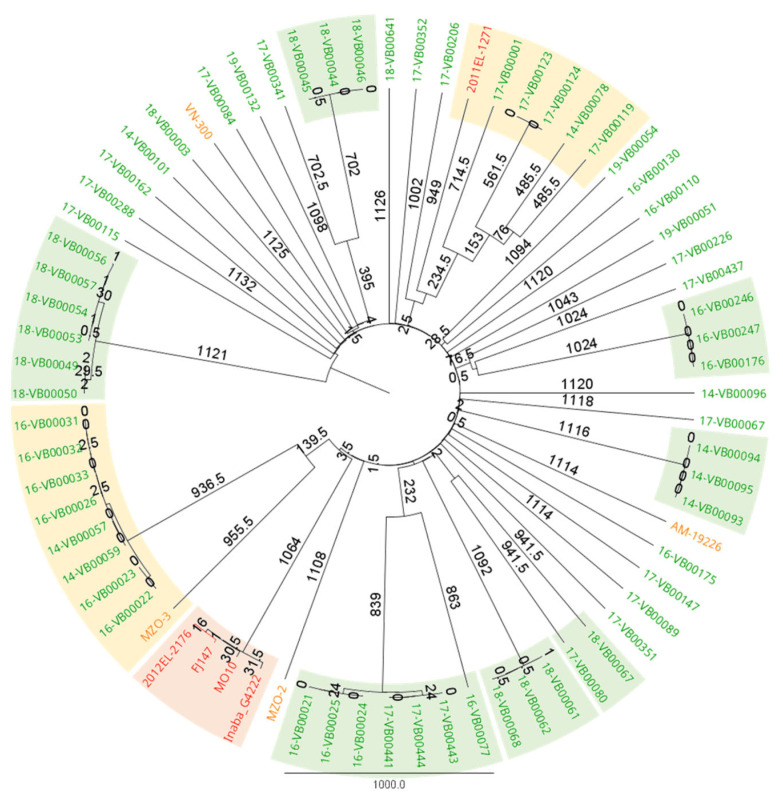
cgMLST analysis of seafood-associated NOVC, clinical NOVC, and pandemic O1/O139 *V. cholerae* strains. Seafood-associated NOVC are labeled in green, clinical NOVC are labeled in orange, and pandemic O1/O139 *V. cholerae* are labeled in red. A green background shows a clustering of only seafood-associated NOVC. An orange background shows clusters containing clinical NOVC or pandemic O1/O139 *V. cholerae* and seafood-associated NOVC. A red background shows a clustering of only pandemic O1/O139 *V. cholerae*. The clusters were defined based on a clustering threshold ranging from 1 to 1000 allelic differences to identify a phylogenetic relationship according to Liang et al., 2020 [42]. The minimum spanning tree was created using grapetree [28] and visualized using Geneious software (v2022.1.1).

The initial screening of food isolates was achieved via PCR using the gene-specific primers of *ompW* for *V. cholerae* species confirmation based on the method of Nandi et al., 2000 [39]. Primers detecting *ctxA* [40] and gene fragments of *rfbO*1 and *rfbO*139 [41] were then used to identify the cholera toxin, as well as the O1 and O139 serogroups. All strains are positive for *ompW* and negative for the other three target genes, which confirms that the collected strains are NOVC. In order to acquire a clear view of the sub-species population level of our NOVC seafood isolates and the relationships between different strains, cgMLST was performed based on the method in [42]. The minimum spanning tree, as shown in Figure 1, exhibits two highlighted clusters (shown in orange). These clusters represent the grouping of five seafood-associated NOVC with an O1 serotype (2011EL-1271) and the grouping of eight seafood-associated NOVC with a clinical NOVC (MZO-3). There is also a cluster comprising only *V. cholerae* of serotypes O1 and O139 (shown in red), as well as six clusters that exclusively encompass seafood-associated NOVC (shown in green).

**Figure 2:**
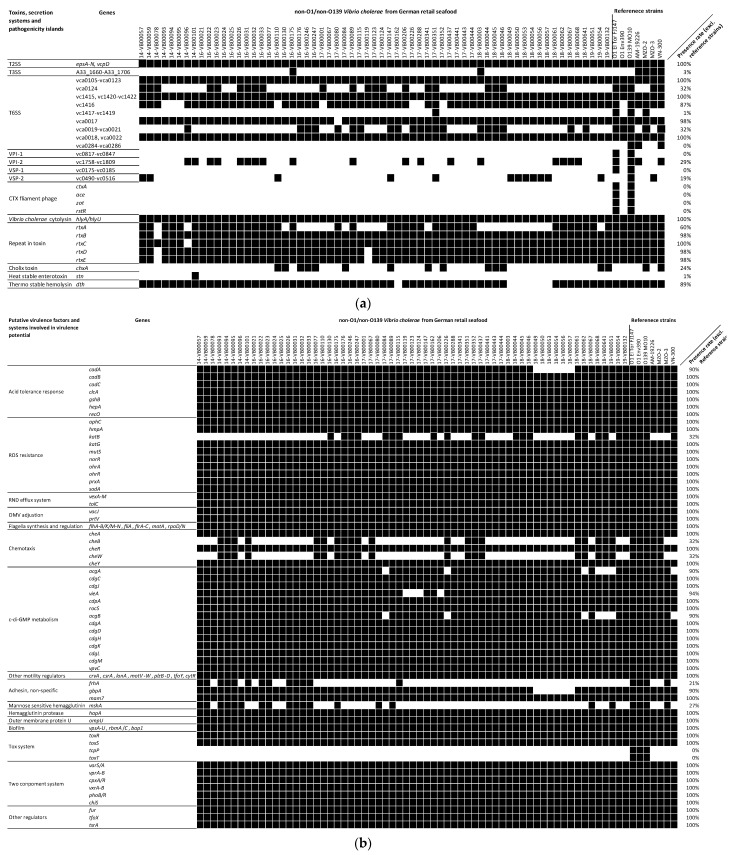
(**a**). The presence/absence of toxin genes, genes involved in secretion systems, and genes included in pathogenicity islands of seafood-associated NOVC (*n* = 63) based on WGS data. Black boxes represent gene presence, and white boxes represent gene absence. Detailed information about the genes can be found in Table S3. (**b**). The presence/absence of genes that play roles as putative virulence factors or genes involved in systems that play roles in the virulence of seafood-associated NOVC (*n* = 63) based on WGS data. Black boxes represent gene presence, and white boxes represent gene absence. Detailed information about the genes can be found in Table S3.

Genotypic results were acquired by comparing the virulence-associated genes (Figure 2a,b and Table S3) of positive control strains to the collected seafood-associated NOVC; the results are shown in Figure 2a,b.

All tested seafood-associated NOVC are positive for most genes conferring adaption in host environments. In summary, genes related to acid tolerance, reactive oxygen species (ROS) resistance, resistance nodulation cell division (RND) efflux systems, and outer membrane vesicle (OMV) regulators were identified in all strains, including reference strains. However, other genes were found in only parts of the seafood-associated NOVC strains studied. These include the lysine decarboxylation mediator *cadA* (90% presence rate) and the catalase encoding gene *katB* (32%). Two genes of the chemotaxis system, namely *cheW* and *cheB*, acting as transporters between chemical receptors and the stabilizer of chemical noise, have a 32% prevalence in the investigated seafood-associated NOVC. Nevertheless, all strains including reference strains harbor other chemotaxis genes (*cheA*, *cheY*, and *cheR)*. The two c-di-GMP modulators, namely *acgAB* and *vieSAB*, were absent in six and four strains of the seafood-associated NOVC, respectively. However, other genes with putative functions in biofilm formation and motility were present in all samples, including reference strains. To identify possible motility and biofilm deficiency in the seafood-associated NOVC based on the absence of genes that have not been considered or are not known so far, we performed in vitro motility and biofilm formation tests.

Three non-specific adhesins, namely *gbpA* (90% prevalence), *mam7* (100% prevalence), and *frhA* (21% prevalence), were identified in the seafood-associated NOVC. Another colonization-involved gene, namely *mshA*, which is the MSHA pili-encoding gene, was also present in 27% of the seafood-associated NOVC. Both VPI-1 and VSP-1 are absent in these isolates, while several ORFs of the two other pathogenicity islands, namely VPI-2 and VSP-2, are present in 29% and 19% of the seafood-associated NOVC, respectively. Although none of the seafood-associated NOVC possessed a full VPI-2, we observed the occurrence of the integrase-, sialidase-, and neuraminidase-encoding segments (vc1758, vc1773-vc1783, and vc1784) among those VPI-2 positive strains [43].

All tested seafood-associated NOVC are negative for cholera related genes, including the CTX phage (*ctxA*, *ace*, *zot*, *rstA*, and *rstB*), *tcpA*, and *toxT*. As for the secretion systems, all samples contained T2SS, while two strains (16-VB00175 and 18-VB00003) possessed whole T3SS when using the *V. cholerae* strain AM-19226-derived T3SS clusters as references (GenBank ID: AATY02000004 and AATY02000003). While most of the T6SS related genes were present in all strains, vca0124 was detected in 32% of strains. The *vgrG*1 encoding gene in auxiliary cluster 1 with actin-crosslinking activity (vc1416) was detected in 87% of strains. The *vasX* encoding gene in auxiliary cluster 2 with pore formation effect on bacterial cells (vca0020) was detected in 68% of strains. Only auxiliary cluster 3, i.e., vca0284 to vca0286, was absent in all strains. The detachment determining factor encoding gene *hapA* was found in all samples. The prevalence rate of *hlyA* (together with its direct regulator *hlyU*) was 100%, which is much higher than of the other additional toxin encoding genes for the thermostable hemolysin (*dth*; 89%), the multifunctional autoprocessing RTX toxins (*rtxA*-*rtxE*; 60%), the cholix toxin (*chxA*; 24%), and the heat-stable enterotoxin (*stn*; 1%).

### 3.2. Phenotypic Characterization

Phenotypic tests were designed to verify the bioinformatic data and further explore the virulence potential of the collected strains. Table 1 shows the percentage of seafood-associated NOVC isolates that can form a biofilm; are resistant to human blood serum, hemolyze human, and sheep erythrocytes; and are motile. Over 90% of the investigated NOVC can form strong biofilms at 37 °C (Table 1 and Table S4). Serum resistance was examined through a gradient mixture of human serum and standard protein–peptone solution. O1 strains were sensitive to human serum under our experimental conditions [7]. For NOVC, five strains were able to withstand 60% human serum, nine strains were able to withstand 40% human serum, and the remaining 49 strains were sensitive and grew only in 0 to 20% human serum. All strains could lyse sheep and human erythrocytes with variation in the hemolysis ring. Five strains have the same hemolytic zone as the control O1 El Tor strain and are shown in Table 1 and S4 to possess strong hemolytic activity: 17-VB00123, 17-VB00124, 18-VB00049, 18-VB00056, and 18-VB00057.

As for the motility characteristics on soft agar plates (Table 1 and Table S4), 92% of strains have medium-to-strong motility. Almost all strains showed a medium to high motility at 37 °C.

### 3.3. Antimicrobial Resistance Profile

In total, the non-wildtype phenotypes of seven antibiotics were noticed in the NOVC collection: AMP (11%), CIP (2%), COL (87%), FOX (2%), IMIPEN (78%), NAL (5%), and TMP (6%). Although the official *Vibrio* spp. breakpoint is not available for COL, NAL, and TMP, strains that could grow under the maximum MIC value were considered as resistant [44]. No resistance against AZI, CEFEPI, CHL, ERTAPE, FOT, GEN, MERO, SMX, TAXCLA, TAZ, TAZCLA, TEMOCI, TET, and TGC was observed in any of the investigated strains. Five seafood-associated strains were found to be simultaneously resistant to three antibiotic classes (beta-lactams: IMIPEN; polymyxins: COL; and sulphonamides: TMP): 16-VB00021, 16-VB00024, 16-VB00025, 17-VB00441, and 19-VB00051.

**Figure 3:**
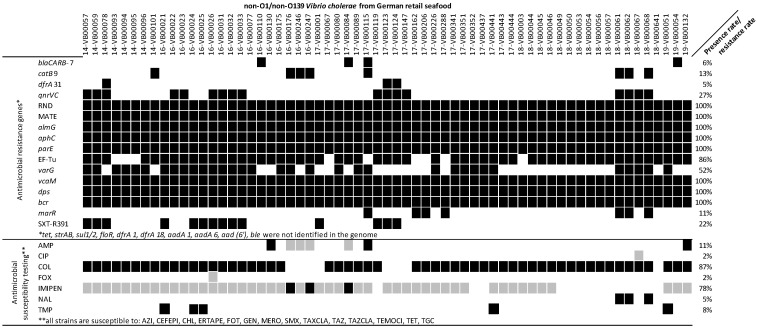
Antibiotic resistance pattern of NOVC strains (*n* = 63) at genetic and phenotypic level. SXT/R391: conserved region of SXT/R391. *bcr*: Bcr/CflA family efflux transporter. *dps*: member of ferritin-like diiron-carboxylate proteins encoding gene. *vcaM*: ATP binding cassette encoding gene. *varG*: VarG beta-lactamase Subclass B1 encoding gene. EF-Tu: elfamycin resistance gene. *parE*: fluoroquinolone resistance gene. *almG*: lipid A acyltransferase encoding gene. MATE: multidrug and toxic compound extrusion pump encoding gene. *qnrVC*: quinolone resistance gene. *dfrA*31: trimethoprim resistant dihydrofolate reductase encoding gene. *catB*9: chloramphenicol acetyltransferase encoding gene. *blaCARB*-7: beta-lactamase encoding gene. In genetic result, black boxes represent gene presence, and white boxes represent gene absence. In phenotypic result, black boxes represent resistance, grey boxes represent intermediate, and white boxes present susceptibility. Detail information about breakout points is shown in Table S2.

Moreover, all strains are positive for the multidrug and toxic compound extrusion pump (MATE) efflux system (*vcmA*, *vcmB*, *vcmD*, *vcmH*, *vcrM*, and *vcrN*), the ABC transporter (*vcaM*), and the genes involved in antibiotic resistance: *parE*, *dps*, *almG*, and *bcr*. The quinolone resistance factors *qnrVC*4, *qnrVC*5 and *qnrVC*7 (27% prevalence), the Ambler class B metallo-beta-lactamase gene *varG* (52% prevalence), the CARB beta-lactamase *blaCARB*7 (6% prevalence), the chloramphenicol acetyltransferase *catB*9 (13% prevalence), the elfamycin resistance gene EF-Tu (86% prevalence), and the dihydrofolate reductase dfrA31 (5% prevalence) were also detected in the strain set. The resistance genes for tetracycline (*tet*), florfenicol (*floR*), streptomycin (*strA/B*), sulfamethoxazole (*sul1* and *sul2*), bleomycin (*ble*), and aminoglycoside (*aadA*1, *aadA*5, and *aac*(6′)) were absent in all seafood-associated NOVC. Fourteen strains had a conserved region of ICE SXT/R391 (Figure 3), which represents the presence of MGE. However, only a few segments were identified, compared to the 60 segments identified in SXT/R391 in *V. cholerae* [45].

## 4. Discussion

In this study, the virulence and antimicrobial resistance potential of seafood-isolated NOVC strains were investigated through genotypic and phenotypic approaches. In cgMLST, thirteen seafood-isolated NOVC have close relationships with clinical NOVC (Figure 2), indicating a similar core genome between seafood isolated and clinical NOVC.

### 4.1. Adaption between Environment and Host

Apart from toxin-producing genes and genes strongly associated with them, which have been the focus of previous studies of the virulence potential of NOVC [7,46], other genes not directly related to toxicity also play roles in the infection process [47]. Acid tolerance-related genes are present in most seafood-isolated NOVC, while six strains are negative for *cadA*, which could encode lysine decarboxylase and play important roles in host gastrointestinal surveillance [48]. As for the ROS resistance, 32% of *katB* and 100% of other relevant genes were detected in the strain collection. The catalase-encoding gene *katB*, associated with H_2_O_2_ reduction activity [49], is absent in some strains. *katB*, in *V. cholerae*, seems to make a limited contribution to catalase activity when *katG* (an additional catalase-encoding gene) is active [50].

Other studies have highlighted the relationship between the motility and toxicity of *V. cholerae*. Motility could contribute to localization on biotic and abiotic surfaces, including the successful colonization of the gut, mucus penetration, and further systemic infections [51]. Genes required for flagella synthesis and regulation were detected in all NOVC considered in this study. Potential motility-modulating genes are also located in all the analyzed NOVC except *acgAB*, i.e., a pair of genes in c-di-GMP (an important life-mode switching signal) synthesis [52]. The subsequent motility assay revealed that most NOVC are motile, whereas the less motile strains could not be explained by the presence or absence of specific motility-modulating genes (see Figure 2b). All strains are more motile at 37 °C than at 25 °C, which is consistent with other studies defining increased c-di-GMP levels at low temperature as a modulator of life adaption [53]. This study and other studies show that O1 strains can be less motile than NOVC [54].

In addition to motility, the formation of biofilms has advantages in the infection process, as biofilms are an adaptation not only to harsh environmental conditions but also to host conditions [55]. The genes required for polysaccharide and essential protein recruitment were present in all strains, whereas the absence of *acgAB* and *vieSAB* might have an impact through c-di-GMP alteration [52]. The results of the in vitro biofilm assay showed that 90% of strains form strong biofilms, highlighting their adaption ability [56]. On the other hand, there was one strain which was not able to build a biofilm and five strains with medium biofilm formation ability, suggesting that these strains might be more susceptible to unfavorable environment and host conditions because they are not protected by biofilms [55].

The chemotaxis system might be relevant to chemical sensing and the following reaction. This system might be incomplete in our investigated NOVC strains because of the basic gene in the chemotaxis working frame, namely *cheW*, was only identified in 32% of strains [57]. Early reports mentioned that chemotaxis mutant *V. cholerae* strains have different moving behaviors, leading to them having a wider distribution in intestines than wild-type strains [58].

### 4.2. Adherence and Toxin Production Ability

The attachment and proliferation on intestinal epithelial cells is the prerequisite of toxin production [47]. We noticed that several non-specific adhesins were missing in our seafood isolates, such as *gbpA* and *frhA*, indicating the intestinal epithelial cell attachment deficiency for the respective strains [59,60]. VPI-1, the critical colonization determinant, was missing in all isolates. The complete T3SS of the reference strain AM-19226 (a clinical NOVC) was identified in two strains, namely 16-VB00175 and 18-VB00003, and those two strains were closely related to clinical NOVC strains with T3SS (AM-19226 and VN-300), according to the cgMLST result (Figure 1). This result suggests that these two strains might have better intestine adherence and proliferation ability during infection compared to other strains [29,61]. According to a large-scale genomic search for *V. cholerae*, all NOVC with human infection record are T3SS-positive [62]. We also observed that two pathogenicity islands (PIs) were partially (VPI-2, VSP-2) present, which unveiled the putative function of sialic acid utilization (VPI-2) and di-nucleotide molecule production (VSP-2) for relevant strains [43,63]. VPI-1, which might have impact on environment adaption, was completely missing in our collection [64]. However, NOVC could grow and show infectivity without these three PIs in spite of their contributive functions in the infection process [29]. The large cluster of T6SS was identified in all strains, while the auxiliary clusters 1 and 2 (encoding *vgrG*1 and *vasX* respectively) were partially present, which is similar to the clinical NOVC gene-screening result from Arteaga et al., 2020 [62]. Previous works suggested that *vgrG*1 has a toxic effect on eukaryotic cells by forming pores or disrupting cellular structures, *vasX* has the potential to disrupt the gut microbiome and hemostasis, and *vgrG*3 plays a role in antibacterial activity that allows *V. cholerae* to establish itself as the dominant species [65]. Auxiliary cluster 3 of T6SS (vca0284 to vca0286) is missing in all NOVC strains, which could encode amidase TseH and degrade the peptidoglycan cover of bacteria [66]. Genes with regulation effect on T6SS were also found in all NOVC strains (*tfoX*, *tfoY*, and *tsrA*), indicating the proper expression of T6SS [65].

The filamentous CTX phage was not detected in any of the analyzed seafood-associated strains, which is consistent with the characteristics of most NOVC [46]. All strains are positive for the hemolysin *hlyA* and its regulator *hlyU*, whose functionality have been demonstrated via blood agar hemolysis for sheep and human blood [67]. Other researchers also found the high occurrence rate of *hlyA* in NOVC [46]. After the consumption of contaminated seafood, a transition of motile NOVC into the bloodstream through the portal vein or intestinal lymphatic system is possible [68]. In this study, 14 NOVC strains were identified as having medium-to-high resistance against human serum, showing their ability to survive in human blood and cause bacteremia. For 13 of these serum resistant strains, a correlation with medium-to-high biofilm formation ability was identified (Table S4). King et al., 2009, reported this relationship in *Acinetobacter baumannii*, a Gram-negative pathogen, suggesting that either the better biofilm formation results from serum resistance or the biofilm substance is beneficial for serum resistance.

In addition, several other toxin-encoding genes were detected, including *chxA*, *stn*, and *rtxA*. The heat-stable enterotoxin-encoding gene *stn* was only present in one strain: 14-VB00101. The ADP-ribosylation factor, namely cholix toxin *chxA*, was detected in 24% of strains, which could induce mitochondrial dysfunction and cytoskeletal rearrangement. The toxicity of these accessory toxins was previously confirmed via a mouse model [69,70,71]. Furthermore, *hlyA*, *rtxA*, *chxA*, and T3SS were detected in clinical NOVC isolated from German and Austrian patients by Schirmeister et al., 2014 [7], while higher *hlyA* prevalence was noticed in our strain collection. The fact that these virulence-associated genes are also present in NOVC isolated from German retail seafood in this study confirms the assumption that these strains have a pathogenic potential.

### 4.3. Antimicrobial Resistance Ability

Fourteen strains with conserved region of SXT/R391 were compared to a SXT/R391 collection [45], and the presence of fragments was confirmed. Moreover, a whole integron (integrase *intI* and recombination site *attI/attC*) was detected in 60% of the seafood-associated NOVC via IntegronFinder [72]. These results suggest the potential for the HGT transition of isolated NOVC in coastline seawater [21]. Antibiotic resistance could be simultaneously shown against three antibiotic classes (beta-lactams, for example, penicillin, cephalosporin, and carbapenem; polymyxins; and sulfonamides) for five seafood-associated NOVC (16-VB00021, 16-VB00024, 16-VB00025, 17-VB00441, and 19-VB00051). Relevant gene distribution could explain some results from antibiotic in vitro assays, including *blaCARB* presence and AMP resistance [73,74], *almG* presence and COL resistance [75], *qnrVC*s presence and quinolone (CIP and NAL) resistance [76], *varG* and IMIPEN resistance [77], and *dfrA*31 presence and TMP resistance [78]. However, AST result cannot be fully explained by genetic information: the COL resistance rate is 87%, while all NOVC strains used in this study contain *almG*; *blaCARB* was not found in the three AMP resistant strains; and all *catB*9 harboring strains have no in vitro resistance to CHL. The reason for this trend might be gene variation, while incomplete databases could lead to discrepancies, and there might be other genes that relativize the phenotype [19]. Doxycycline (a second-generation TET), AZI, and CIP are recommended for cholera treatment by the CDC. In this study, only resistance against CIP (2%) was detected.

## 5. Conclusions

In summary, the putative virulence of seafood-associated NOVC was confirmed, in particular by the presence of virulence genes, such as *hlyA*, *rtxA*, *chxA*, and the T3SS. In total, 13 of 63 NOVC originally isolated from seafood products clustered together with clinical *V. cholerae* strains in cgMLST, indicating a possible relationship between them. A strong capacity for biofilm formation, motility, and hemolytic activity were confirmed via phenotypic experiments. Antibiotics recommended by the CDC for cholera treatment are effective against infection with seafood-associated NOVC. Only 2% of strains are resistant to ciprofloxacin.

Nevertheless, seafood-associated NOVC have the genetic and phenotypic potential to cause human infections through the consumption of contaminated seafood and should be included in future seafood surveillance.

## Figures and Tables

**Table 1:** Phenotypic characterization of seafood-associated NOVC (*n* = 63).

Classification	Biofilm Formation	Serum Resistance	Hemolytic Activity	Motility
Strong	90% (*n* = 57)	8% (*n* = 5)	8% (*n* = 5)	52% (*n* = 33)
Medium	8% (*n* = 5)	14% (*n* = 9)	92% (*n* = 58)	40% (*n* = 25)
Weak	0% (*n* = 0)	22% (*n* = 14)	0% (*n* = 0)	8% (*n* = 5)
None	2% (*n* = 1)	56% (*n* = 35)	0% (*n* = 0)	0% (*n* = 0)

## Data Availability

The assembled genomes of the 64 NOVC strains can be found in the NCBI database under the corresponding BioSample numbers, which are listed in Table S5.

## Supplementary Materials

The following supporting information can be downloaded via this link: https://www.mdpi.com/article/10.3390/microorganisms11112751/s1, Table S1: Bacterial strains; Table S2: Antimicrobial resistance data; Table S3: Detailed information on genes/gene clusters and their functions and references; Table S4: Results of phenotypic assays; Table S5: BioSample numbers of the assembled genomes of each bacterial strain.
